# Incidence of Elevated Aminotransferases With or Without Bilirubin Elevation During Treatment With Immune Checkpoint Inhibitors: A Retrospective Study of Patients From Community Oncology Clinics in the United States

**DOI:** 10.7759/cureus.24053

**Published:** 2022-04-11

**Authors:** Christopher Kim, Shao Zhu, Hosein Kouros-Mehr, Sophia Khaldoyanidi

**Affiliations:** 1 Center for Observational Research, Amgen Inc., Thousand Oaks, USA; 2 Biostatistics, Simulstat Inc., San Diego, USA; 3 Global Development, Amgen Inc., Thousand Oaks, USA; 4 Early Development, Amgen Inc., Thousand Oaks, USA

**Keywords:** retrospective study, immune-checkpoint inhibitors, hyperbilirubinemia, elevated aminotransferases, electronic health records

## Abstract

Introduction

The elevation of aminotransferase levels is regarded as an indicator of hepatocellular injury. The objective of this study was to describe real-world incidence of elevated aminotransferase levels with or without bilirubin elevation among patients treated with immune checkpoint inhibitors (ICIs) for solid tumors.

Methods

This retrospective cohort study used an electronic health record database representing > 1.5 million active United States (US) cancer patients and included patients diagnosed with any cancer between January 1, 2014 and March 31, 2019, and treated with one or more ICIs such as ipilimumab, tremelimumab, nivolumab, pembrolizumab, atezolizumab, durvalumab, and avelumab. The frequency, onset, duration, management of grade ≥ 3 elevation of aminotransferase levels with or without bilirubin elevation events, progression rate from isolated elevation of aminotransferase levels (IAT) to elevated aminotransferase levels with elevated bilirubin (ATWB), and mortality were described.

Results

Overall, 69,140 patients received 85,433 treatment courses. A total of 1,799 (2.11%) IAT and 441 (0.52%) ATWB events were observed during treatment courses. The median onset was 51 and 42 days for IAT and ATWB, respectively, across treatment courses, and the median duration of both was approximately seven days. Approximately 5% (n=96) of IAT events progressed to ATWB in a median time of 11 days. The proportion of patients who received corticosteroids after elevated aminotransferase levels with or without bilirubin was ~37% (n=671/1,799 of IAT and n=147/441 of ATWB) and ~8% discontinued ICI treatment (n=118/1,799 of IAT and n=43/441 of ATWB). About 46% (n=68/147) of ATWB and and 25% (n=172/671) of IAT events treated with steroids led to death within 45 days. Similarly, 49% (n=21/43) of ATWB and 35% (n=42/118) of IAT events leading to treatment discontinuation led to death within 45 days.

Conclusions

Real-world data from oncology clinics in US suggest low incidence of grade ≥ 3 elevated aminotransferase levels with or without bilirubin elevation following treatment with ICIs. In most cases, ICI treatment was not discontinued and management of elevated aminotransferases consisted of corticosteroid treatment in one-third of cases.

## Introduction

Drug-induced liver injury (DILI) is caused by exposure to various drugs and other xenobiotics [1] and is a leading cause of acute liver failure in the United States (US) [2,3]. Drugs across nearly all therapeutic classes can cause DILI, resulting in discontinuation of therapy [4]. DILI is monitored and diagnosed based on elevations in levels of serum biomarkers, such as alanine aminotransferase (ALT) and aspartate aminotransferase (AST; collectively known as aminotransferases [AT]), alkaline phosphatase (ALP), and total bilirubin [5], and the severity of DILI is graded based on Common Terminology Criteria for Adverse Events (CTCAE) [6,7]. Elevation of AT levels in the absence of other clinical laboratory abnormalities in asymptomatic patients has been reported previously [8]. The association between hepatocellular injury and liver dysfunction has been emphasized by Hy’s law, which states that elevation of AT levels (more than three times upper limit of normal [ULN]) accompanied with bilirubin elevation (more than two times ULN) in the absence of ALP elevation (less than two times ULN) was likely to cause the death of a patient, whereas an elevation of AT levels alone may be less specific in detecting serious DILI [9,10]. Hence, a recognition of the drugs in general oncology practice with a potential to cause DILI and methods of mitigation, including treatment discontinuation, is of paramount importance.

Immune-mediated hepatotoxicity is a form of indirect hepatotoxicity caused by immune checkpoint inhibitors (ICIs), including molecules that block programmed cell death 1/programmed cell death ligand 1 (PD-1/PD-L1) and cytotoxic T-lymphocyte-associated protein 4 (CTLA-4) pathways [11-14]. In addition to inflammatory reactions caused by the release of cytokines, the blockade of these pathways may also stimulate autoreactive T-cells, leading to local inflammatory reactions or autoimmune injuries in various organs, including the liver [12,15]. The management of DILI depends on its severity and comprises various therapeutic approaches, including prompt discontinuation of the causative drug and administration of either corticosteroids such as prednisone and methylprednisolone, or immunosuppressants such as azathioprine and mycophenolate mofetil [16,17]. In several clinical trials involving immunotherapeutic agents, elevation of AT levels was reported as a common adverse event, indicating hepatic injury [12,18-20]. To date, real-world evaluations of elevated AT levels with or without elevation of bilirubin associated with ICIs have not been widely reported.

The purpose of this retrospective, observational, and multicenter study was to examine the frequency, duration, progression in isolated elevation of aminotransferase levels (IAT; no bilirubin elevation), and elevated AT levels along with elevated bilirubin (ATWB). Additionally, the frequency of ICI treatment discontinuations and intervention with corticosteroids after occurrence of elevated AT levels with or without bilirubin elevations observed in this study were described [21]. Finally, we also examined death occurring within 45 days of IAT and ATWB events, treatment discontinuations, and steroid treatment.

## Materials and methods

Data sources

This was a retrospective cohort study that used oncology electronic health record (EHR) data contained in Amgen’s Oncology Services Comprehensive Electronic Records (OSCER) database generated from Flatiron Health (New York, NY, USA). OSCER represents a longitudinal, demographically and geographically diverse database with data from over 250 cancer clinics representing over 1.5 million active patients in the US treated at community-based hematology or oncology practices and from three academic centers in the US. Patients represent all payer types (commercial, Medicare, Medicaid, self-pay, and other). The de-identified patient-level data include all structured data elements from the EHR at the oncology clinic (diagnostic details, laboratory values, and prescribed drugs) [22]. The Institutional Review Board of each oncology practice approved the collaboration and the contribution of data to this large longitudinal EHR database. Informed patient consent was waived as per the US framework for retrospective noninterventional studies. Individual patient-level data were protected against breach of confidentiality in line with the regulations developed by the Health Insurance Portability and Accountability Act Security Rule from the US Department of Health and Human Services [7].

Cohort identification

The study included patients diagnosed with solid tumors and treated with ICIs, such as ipilimumab (Yervoy®), tremelimumab (not approved), nivolumab (Opdivo®), pembrolizumab (Keytruda®), atezolizumab (Tecentriq®), durvalumab (Imfinzi®), and avelumab (Bavencio®) from January 1, 2014 to March 31, 2019. Patients with valid numerical value and populated liver function tests (LFTs), such as tests to determine levels of ALT, AST, and bilirubin using blood samples, at baseline and following drug administration were included. Patients with > grade 2 LFT elevations at baseline were excluded from the study.

The index date and beginning of follow-up was the first date when a treatment course with any of the seven ICIs was initiated. The baseline period to define all characteristics of patients was up to 180 days prior to index date of the first treatment course (median: 1 day, range: 0-14 days; mean: 13.38 days, SD: 26.53). Cancer diagnosis was the most recent to the initiation date of the treatment course. The follow-up period for each patient consisted of the duration between the date of the first ICI administration and 45 days after administration of the last ICI based on the median time to livery injury [23,24]. For patients with more than one treatment course with an ICI, each course was considered as an independent observation for this study. Patients could undergo more than one treatment course, which was terminated if treatment was not administered for 45 days. This treatment pattern was applicable to all ICIs, including ≤ three ipilimumab doses. In the circumstances where patients received ≥ four doses of ipilimumab, the treatment course was terminated if treatment was not administered for 95 days. The patients were considered as receiving combination therapy for treatment of solid tumors if they received ≥ two ICIs such as CTLA-4, PD-1, and PD-L1 inhibitors at least once in an overlapping dosing schedule. A switch to a new treatment course occurred when patients who were treated with multiple administrations of a single ICI in the past 30 days received treatment with another ICI, without additional administration of the first ICI. Finally, patients treated with an ICI in conjunction with any other antineoplastic drug were classified as a separate ICI plus antineoplastic category.

Outcome measures

Events of interest, including the highest value of ALT, AST, and bilirubin, were described in each follow-up period beginning on the first day of the administration of a new treatment course. The highest value was assigned a grade based on CTCAE version 4.0.3. In this study, elevated levels of ALT (ULN: 55 U/L, grade 3: >5.0x ULN), AST (ULN: 48 U/L, grade 3: >5.0x ULN), and bilirubin (ULN: 1.2 mg/dL, grade 3: >3.0x ULN) were the events of interest. An IAT event was defined as ≥ grade 3 elevations in ALT or AST levels with ≤ grade 2 bilirubin levels. An ATWB event was defined as ≥ grade 3 elevations in ALT or AST levels and ≥ grade 3 bilirubin levels. The duration of elevation was defined as the period from the first identification of elevation until the next laboratory test in which < grade 3 elevation in ALT or AST levels was recorded. If only one test panel indicated elevated results, the duration of elevation was imputed as the median point between the single elevated test and the normal test dates. Treatment discontinuation after elevated AT levels occurred if treatment was discontinued within 15 days of an event involving elevation in AT levels. If a patient received prednisone or dexamethasone within 15 days of resolution of an event involving elevation in AT levels, it was termed as treatment with a corticosteroid. The exact day of the patient’s death was not available in the database and is automatically set to the 15th day of the month in which death occurred. Thus, the outcome of death was estimated up to 15 days prior to or after 45 days relative to an event.

Statistical analysis

Descriptive analysis was used to summarize patient demographics and baseline disease characteristics. Descriptive statistics on continuous data included medians, standard deviations, and ranges for reporting duration and onset of events involving elevation of AT levels with or without bilirubin events. Elevated AT levels with or without bilirubin event counts were descriptively presented as proportions. Univariate analyses were used to compare occurrence and durations of IAT and ATWB events across different treatment classes. Chi-square for categorical data and rank sum for medians were used for comparisons, and P values less than 0.05 were considered significant.

## Results

Patient characteristics

Patient disposition is shown in Table 1 and patient and disease characteristics are shown in Table 2.

**Table 1:** Patient disposition ALT, alanine aminotransferase; AST, aspartate aminotransferase; N/A, not applicable

	Patients	Treatment courses
Treated with immunotherapies for solid tumor and hematologic cancers in study period 2014-2019	70,605	N/A
Initiated treatment with immunotherapy in 2014 with no related treatment 180 days prior	70,534	N/A
Treatment with one of the treatment courses	70,534	87,439
Age ≥ 18 years at the index date of a treatment course	70,528	87,342
Excluded due to benign tumor or missing cancer diagnosis prior to initiation of a treatment course	69,800	86,449
Excluded due to grade 3 or 4 AST/ALT elevation within 30 days prior to initiation of a treatment course	69,448	85,799
Patients with solid tumor	69,140	85,433

**Table 2:** Patient characteristics Data represented as n (%); *Combination of any two or more CTLA-4, PD-1, and PD-L1 inhibitors; †Any other antineoplastic treatment not belonging to the class of ICIs specifically listed. CTLA-4, cytotoxic T-lymphocyte–associated protein 4; ECOG PS, Eastern Cooperative Oncology Group performance status; ICI, immune checkpoint inhibitor; ICI Comb, combinations of ICIs; PD-1, programmed cell death 1; PD-L1, programmed cell death ligand 1.

	Patients (N = 69,140)	Treatment courses (N = 85,433)
Age at baseline		
18–29	275 (0.40)	382 (0.45)
30–39	937 (1.36)	1,210 (1.42)
40–49	2,810 (4.06)	3,502 (4.10)
50–59	10,556 (15.27)	12,897 (15.10)
60–64	9,700 (14.03)	11,719 (13.72)
65–69	11,662 (16.87)	13,902 (16.27)
70–79	23,106 (33.42)	28,022 (32.80)
> 80	11,393 (16.48)	13,799 (16.15)
Race		
Asian	1,140 (1.64)	1,412 (1.65)
Black	4,373 (6.32)	5,389 (6.31)
Hispanic	91 (0.13)	113 (0.13)
Other	8,468 (12.24)	10,488 (12.27)
White	48,891 (70.71)	60,665 (71.01)
Unknown	6,177 (8.93)	7,376 (8.63)
Type of cancer		
Lung	29,217 (42.26)	34,527 (40.41)
Secondary malignancies and metastatic disease	19,967 (28.88)	24,412 (28.57)
Melanoma	5,933 (8.58)	7,962 (9.32)
Kidney	2,738 (3.96)	3,190 (3.73)
Bladder	2,283 (3.30)	2,640 (3.09)
Prostate	357 (0.52)	429 (0.50)
Liver	1,107 (1.60)	1,259 (1.47)
Liver carcinoma	660 (0.95)	739 (0.87)
Other	9,496 (13.73)	11,014 (12.89)
ECOG PS		
Missing	18,076 (26.14)	21,102 (24.70)
0	16,302 (23.58)	19,258 (22.54)
1	26,601 (38.47)	30,794 (36.04)
2	10,563 (15.28)	11,618 (13.60)
3	2,342 (3.39)	2,503 (2.93)
4	146 (0.21)	158 (0.18)
Treatment class		
CTLA-4 inhibitors	2,007 (2.90)	2,526 (2.96)
PD-1 inhibitors	48,295 (69.85)	57,421 (67.21)
PD-L1 inhibitors	6,712 (9.71)	7,608 (8.91)
ICI Comb*	5,025 (7.27)	5,274 (6.17)
ICI plus antineoplastic^†^	12,073 (17.46)	12,604 (14.75)

Data from patients with normal or elevated (grade 1) LFTs at baseline were analyzed in this study. Overall, 85,433 treatment courses were administered to 69,140 patients. A total of 80,339 treatment courses (94.04%) were administered to patients with solid tumors aged ≥ 50 years, of which the highest number of treatment courses were administered to patients aged 70-79 years (28,022; 32.80%). Although the Eastern Cooperative Oncology Group performance status (ECOG PS) was missing at treatment initiation for 18,076 (26.14%) patients, most treatment courses (61,670; 72.18%) were administered to patients with ECOG PS 0-2. At treatment initiation, patients diagnosed with lung and secondary malignancies and metastatic disease were collectively administered a greater number of treatment courses (58,939; 68.99%) than those with other types of solid tumors. Among all drugs, the highest number of treatment courses consisted of PD-1 inhibitors (57,421; 67.21%).

Isolated elevation of aminotransferase levels (IAT) and elevated aminotransferase levels along with elevated bilirubin (ATWB)

Frequency of IAT and ATWB Events

Overall, treatment courses resulted in IAT and ATWB events ranging from approximately 0.99%-6.81% and 0.20%-1.38%, respectively (Table 3).

**Table 3:** IAT and ATWB events Data represented as n (%); p value calculated by chi-square; *Combination of any two or more CTLA-4 inhibitors, PD-1 inhibitors, and PD-L1 inhibitors; †Any other antineoplastic treatment not belonging to the class of ICIs specifically listed. ATWB, elevated AT levels along with elevated bilirubin; CTLA-4, cytotoxic T-lymphocyte–associated protein 4; Gr, grade; IAT, isolated elevation of AT levels; ICI, immune checkpoint inhibitor; ICI Comb, combinations of ICIs; N, total number of events; PD-1, programmed cell death 1; PD-L1, programmed cell death ligand 1.

Treatment class	Treatment courses (%)	IAT			ATWB		
		Gr 3	Gr 4	Total	Gr 3	Gr 4	Total
N (%)	85,433 (100)	1,630 (1.91)	169 (0.20)	1,799 (2.11)	378 (0.44)	63 (0.07)	441 (0.52)
CTLA-4 inhibitors	2,526 (2.96)	69 (2.73)	7 (0.28)	76 (3.01)	14 (0.55)	2 (0.08)	16 (0.63)
PD-1 inhibitors	57,421 (67.21)	949 (1.65)	84 (0.15)	1,033 (1.80)	247 (0.43)	30 (0.05)	277 (0.48)
PD-L1 inhibitors	7,608 (8.91)	71 (0.93)	4 (0.05)	75 (0.99)	14 (0.18)	1 (0.01)	15 (0.20)
ICI Comb^*^	5,274 (6.17)	305 (5.78)	54 (1.02)	359 (6.81)	48 (0.91)	25 (0.47)	73 (1.38)
ICI plus antineoplastic^†^	12,604 (14.75)	236 (1.87)	20 (0.16)	256 (2.03)	55 (0.44)	5 (0.04)	60 (0.48)
p value		<0.0001	<0.0001	<0.0001	<0.0001	<0.0001	<0.0001

A total of 1,799 (2.11%) IAT and 441 (0.52%) ATWB events occurred in 85,433 treatment courses (Table 3), and the treatment courses consisting of ICI monotherapy such as CTLA-4, PD-1, and PD-L1 inhibitors accounted for 1,184 (65.81%) IAT events and 308 (69.84%) ATWB events. A significantly different number of IAT (359; 6.81%) and ATWB (73; 1.38%) events were reported in patients across treatment types (p, chi-square < 0.0001), and one notable finding was combination therapy of two or more ICIs being higher than other treatment courses.

Progression From IAT to ATWB Events

Of 1,799 IAT events reported in treatment courses administered, 96 (5.34%) progressed to ATWB in a median time interval of 11 (1-161) days (Table 4).

**Table 4:** Progression from IAT to ATWB events Data represented as n (%) unless indicated otherwise; p value calculated by rank sum test; *Combination of any two or more CTLA-4 inhibitors, PD-1 inhibitors, and PD-L1 inhibitors; †Any other antineoplastic treatment not belonging to the class of ICIs specifically listed. ATWB, elevated AT levels along with elevated bilirubin; CTLA-4, cytotoxic T-lymphocyte–associated protein 4; IAT, isolated elevation of AT levels; ICI, immune checkpoint inhibitor; ICI Comb, combinations of ICIs; N, total number of events; NA, not applicable; PD-1, programmed cell death 1; PD-L1, programmed cell death ligand 1.

Treatment class	Treatment courses (%)	IAT (A)	ATWB (B)	Progression from (A) to (B)	Days to progression from (A) to (B), mean; median (Min–Max)
N (%)	85,433 (100)	1,799 (2.11)	441 (0.52)	96 (5.34)	21; 11 (1–161)
CTLA-4 inhibitors	2,526 (2.96)	76 (3.01)	16 (0.63)	4 (5.26)	7; 8 (4–10)
PD-1 inhibitors	57,421 (67.21)	1,033 (1.80)	277 (0.48)	59 (5.71)	20; 10 (1–161)
PD-L1 inhibitors	7,608 (8.91)	75 (0.99)	15 (0.20)	2 (2.67)	7; 7 (6–7)
ICI Comb^*^	5,274 (6.17)	359 (6.81)	73 (1.38)	16 (4.46)	23; 15 (6–97)
ICI plus antineoplastic^†^	12,604 (14.75)	256 (2.03)	60 (0.48)	15 (5.86)	31; 13 (1–130)
p value					0.2965

In general, a similar proportion of patients treated with CTLA-4 and PD-1 inhibitors and ICI plus antineoplastic treatment progressed from IAT to ATWB (5.26%-5.86%). Although patients treated with PD-L1 inhibitors experienced a fewer number of IAT events that progressed to ATWB (two; 2.67%), the progression of these events occurred in two patients at six or seven days. Similarly, patients treated with CTLA-4 inhibitors progressed from IAT to ATWB events in a median period of eight (four to 10) days. While not statistically significant, a trend was observed for slower progression of IAT to ATWB (≥ 10 days) for PD-1 inhibitors, ICI combination therapy, and ICI plus antineoplastic drugs than CTLA-4 and PD-L1 (p, rank sum = 0.2965).

Onset and Duration of Events

The median onset of IAT occurred within 51 (1-1,256) days following initiation of a treatment course (Table 5). 

**Table 5:** Onset of IAT and ATWB events *Combination of any two or more CTLA-4 inhibitors, PD-1 inhibitors, and PD-L1 inhibitors; p value calculated by rank sum test; †Any other antineoplastic treatment not belonging to the class of ICIs specifically listed. ATWB, elevated AT levels along with elevated bilirubin; CTLA-4, cytotoxic T-lymphocyte–associated protein 4; IAT, isolated elevation of AT levels; ICI, immune checkpoint inhibitor; ICI Comb, combinations of ICIs; PD-1, programmed cell death 1; PD-L1, programmed cell death ligand 1; SD, standard deviation.

	IAT (days)		ATWB (days)	
	Median (Min–Max)	Mean (SD)	Median (Min–Max)	Mean (SD)
All treatment courses	51 (1–1,256)	87 (114.0)	42 (1–1,256)	77 (118.9)
CTLA-4 inhibitors	63 (1–406)	70 (62.9)	48 (5–126)	50 (34.8)
PD-1 inhibitors	44 (1–1,256)	90 (127.3)	40 (1–1,256)	79 (136.2)
PD-L1 inhibitors	52 (1–518)	78 (97.9)	39 (1–122)	45 (38.1)
ICI Comb*	61 (1–759)	78 (85.8)	49 (1–302)	62 (51.1)
ICI plus antineoplastic^†^	56 (1–748)	92 (107.5)	59 (1–550)	96 (117.2)
p value	0.0986		0.5301	

A non-statistically significant trend for earlier onset of IAT was reported in patients treated with PD-1 inhibitors (44; 1-1,256 days) than that reported in patients treated with other treatment courses with ICIs (p, rank sum = 0.0986). The median onset of ATWB occurred within 42 (1-1,256) days following initiation of a treatment course. Similarly, a non-statistically significant trend for an earlier onset of ATWB was observed in patients treated with PD-L1 (39; 1-122 days) and PD-1 inhibitors (40; 1-1,256 days) than that in patients treated with other treatment courses with ICIs (p, rank sum = 0.5301). Overall, the median onset of IAT and ATWB in patients receiving treatment for solid tumors ranged from 44-63 and 39-59 days, respectively. Furthermore, the median duration of IAT and ATWB events in patients treated with ICIs for solid tumors was approximately seven days (Table 6).

**Table 6:** Duration of IAT and ATWB events p value calculated by rank sum test; *Any two or more of the above therapies (CTLA-4 inhibitors, PD-1 inhibitors, and PD-L1 inhibitors); †Any other antineoplastic treatment not belonging to the class of ICIs specifically listed. ATWB, elevated AT levels along with elevated bilirubin; CTLA-4, cytotoxic T-lymphocyte–associated protein 4; IAT, isolated elevation of AT levels; ICI, immune checkpoint inhibitors; ICI Comb, combinations of ICIs; PD-1, programmed cell death 1; PD-L1, programmed cell death ligand 1; SD, standard deviation.

	IAT (days)		ATWB (days)	
	Median (Min–Max)	Mean (SD)	Median (Min–Max)	Mean (SD)
All treatment courses	7 (0–128)	11.53 (12.61)	7 (0–128)	13.50 (13.49)
CTLA-4 inhibitors	7 (1–122)	12.98 (17.09)	7 (2–54)	13.59 (13.59)
PD-1 inhibitors	7 (0–128)	11.25 (12.16)	7 (0–128)	13.10 (13.57)
PD-L1 inhibitors	7 (1–47)	8.26 (7.93)	7 (2–19)	9.50 (4.45)
ICI Comb*	7 (1–115)	12.93 (13.93)	7 (1–70)	15.97 (15.36)
ICI plus antineoplastic^†^	7 (0–91)	11.18 (11.81)	8 (1–61)	13.35 (11.98)
p value	0.0881		0.8842	

Treatment Discontinuations, Corticosteroid Treatment, and Deaths After IAT

**Figure 1:**
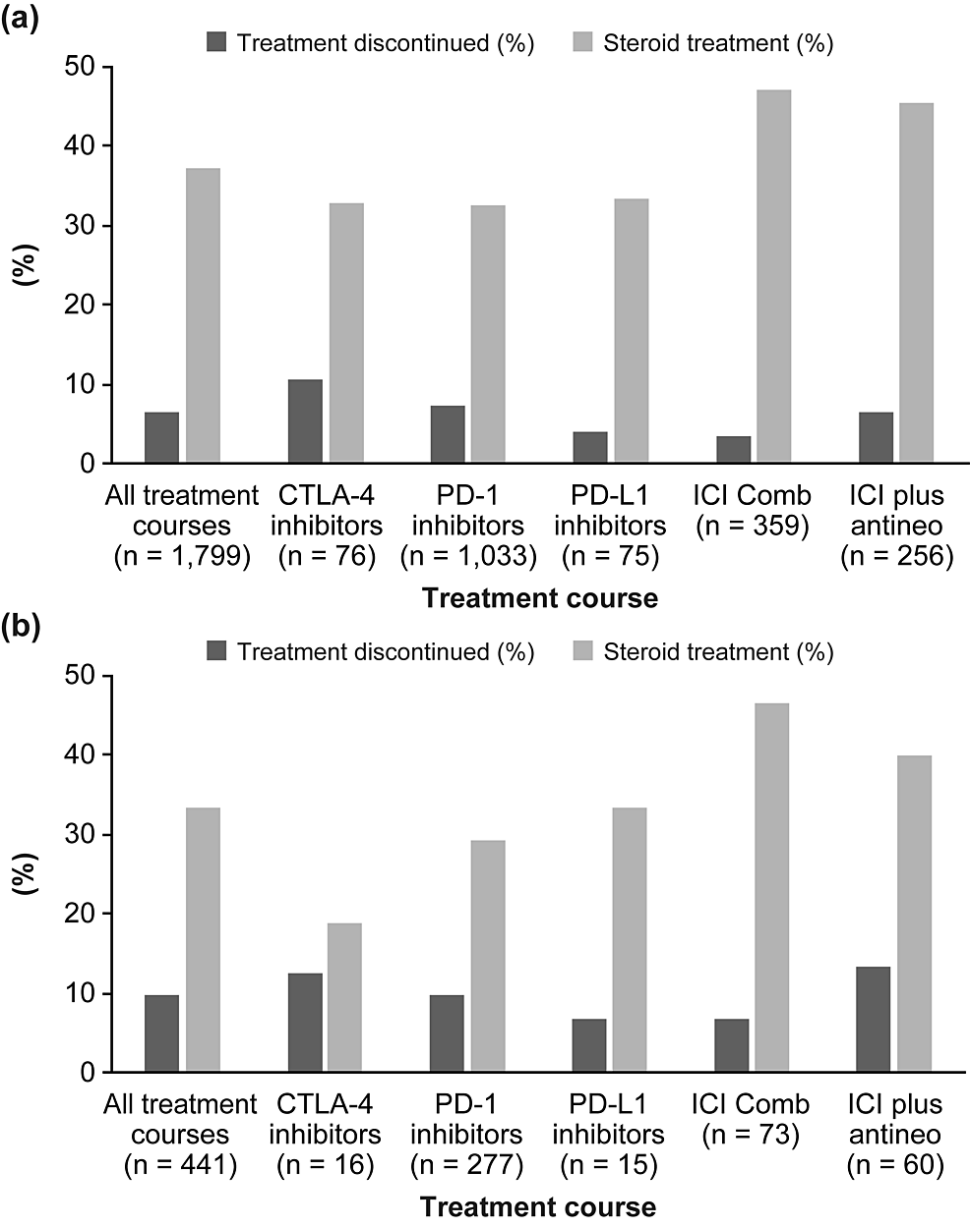
Proportion of treatment discontinuations and steroid treatment in patients with (a) IAT and (b) ATWB events after ICIs ATWB, elevated AT levels along with elevated bilirubin; CTLA-4, cytotoxic T-lymphocyte–associated–protein 4; ICI, immune checkpoint inhibitors; ICI Comb, combination of ICIs; ICI plus antineo, ICI plus antineoplastic treatment; IAT, isolated elevation of AT levels; n, number of events in the treatment group; PD-1, programmed cell death-1; PD-L1, programmed cell death ligand 1.

Overall, among patients experiencing elevations of AT levels with and without bilirubin elevation, the proportion of patients treated with corticosteroids or discontinued treatment courses are presented in Figure 1.

Among all treatment courses administered for solid tumors that resulted in an IAT event, the proportion of patients who received corticosteroids (671; 37.30%) varied largely by the type of ICI treatment with more patients treated with ICI combination receiving corticosteroids (169; 47.08%; p, chi-square < 0.0001; Figure 1a). Overall, a small proportion of patients discontinued ICI treatment (3.62%-10.53%) with a trend toward more patients discontinuing treatment with CTLA-4 inhibitors (eight; 10.53%; p, chi-square = 0.0572; Figure 1a). A small number of patients (40; 2.22%) discontinued treatment courses after IAT and were treated with corticosteroids, including those with grade 3 (37; 2.27%) and grade 4 (three; 1.78%). The median dose of orally or intravenously administered corticosteroids to patients for treatment of IAT was 40 (1-160) mg or 20 (2-1000) mg, respectively.

**Figure 2:**
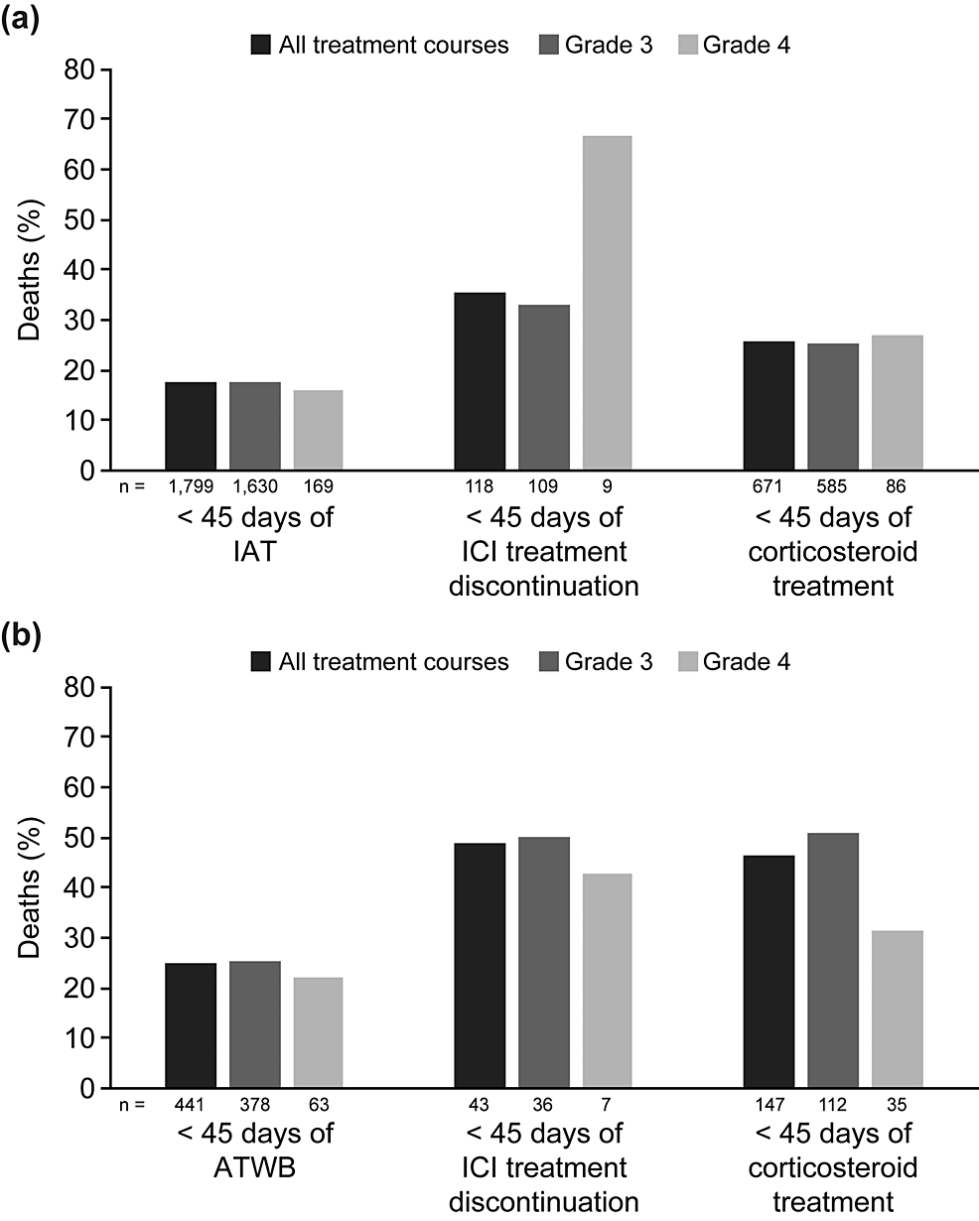
Proportion of deaths associated after (a) IAT and (b) ATWB: overall and grade-wise division ATWB, elevated AT levels along with elevated bilirubin; IAT, isolated elevation of AT levels; ICI, immune checkpoint inhibitors

Overall, 317 (17.62%) patients died within 45 days of occurrence of IAT (Figure 2a).

A similar proportion of deaths was observed in patients with grade 3 (290; 17.79%) and grade 4 (27; 15.98%) IAT (Figure 2a). A significantly greater proportion of deaths within 45 days of occurrence of IAT was observed in patients treated with PD-1 inhibitors (212; 20.52%) than those treated with CTLA-4 inhibitors (six; 7.89%; p, chi-square < 0.0001; not included in tables or figures). None of the patients treated with CTLA-4 inhibitors, PD-L1 inhibitors, or ICI combinations died within 45 days after treatment discontinuation and corticosteroid treatment. Of 671 (37.30%) patients treated with corticosteroids for resolution of IAT, 172 (25.63%) patients died within 45 days (Table 7). A higher proportion of deaths within 45 days in patients treated with corticosteroids was observed with PD-L1 inhibitors (12, 48.00%) vs overall treatment regimens (172; 25.63%; p, chi-square = 0.0850; Table 7).

**Table 7:** IAT: Treatment discontinuation, corticosteroid treatment, and deaths Data represented as n (%); p value calculated by chi-square; *Any two or more of the above therapies (CTLA-4 inhibitors, PD-1 inhibitors, and PD-L1 inhibitors); †Any other antineoplastic treatment not belonging to the class of ICIs specifically listed. CTLA-4, cytotoxic T-lymphocyte–associated protein 4; IAT, isolated elevation of AT levels; ICI, immune checkpoint inhibitors; ICI Comb, combinations of ICIs; n, patients with IAT; PD-1, programmed cell death 1; PD-L1, programmed cell death ligand 1.

(A) Treatment class (n)	(B) Treatment discontinued (% of A)	Treatment discontinued with death < 45 days (% of B)	(C) IAT events treated with corticosteroids (% of A)	IAT events treated with corticosteroids with death < 45 days (% of C)
All (1,799)	118 (6.56)	42 (35.59)	671 (37.30)	172 (25.63)
CTLA-4 inhibitors (76)	8 (10.53)	0	25 (32.89)	3 (12.00)
PD-1 inhibitors (1,033)	77 (7.45)	32 (41.56)	336 (32.53)	96 (28.57)
PD-L1 inhibitors (75)	3 (4.00)	1 (33.33)	25 (33.33)	12 (48.00)
ICI Comb^*^ (359)	13 (3.62)	2 (15.38)	169 (47.08)	31 (18.34)
ICI plus antineoplastic^† ^(256)	17 (6.64)	7 (41.18)	116 (45.31)	30 (25.86)
p value	0.0572	0.0415	<0.0001	0.0850

Treatment Discontinuations, Steroid Treatment, and Deaths After ATWB

Among all treatment courses with ATWB events, the proportion of patients treated with corticosteroids was 33.33% and the proportion of patients who discontinued treatment was 9.75% (Figure 1b). The proportion of patients treated with corticosteroids varied greatly by the type of ICI treatment, with more patients treated with ICI combination receiving corticosteroids (34; 46.58%; p, chi-square = 0.0317; Figure 1b). A small proportion of patients discontinued ICI treatment (6.67%-13.33%) with a trend toward more patients discontinuing treatment with ICI plus antineoplastic agents (eight; 13.33%; p, chi-square = 0.7591; Figure 1b). A small number of patients (14; 3.17%) discontinued treatment courses after ATWB and were treated with corticosteroids, including those with grade 3 (10; 2.65%) and grade 4 (four; 6.35%).

Overall, a total of 109 (24.72%) patients died within 45 days of occurrence of ATWB (Figure 2b). A similar proportion of deaths was observed in patients with grade 3 (95; 25.13%) and grade 4 (14; 22.22%) ATWB (Figure 2b). A greater proportion of patients treated with PD-L1 inhibitors died within 45 days of occurrence of ATWB (seven; 46.67%) than those treated with CTLA-4 inhibitors (one; 6.25%; p, chi-square = 0.0234). Of 147 (33.33%) patients treated with corticosteroids for resolution of ATWB, 68 (46.26%) patients died within 45 days (Table 8). This trend was consistently observed in patients across all treatment courses. The median dose of orally or intravenously administered corticosteroids to patients for treatment of ATWB was 20 (1-100) mg or 20 (4-1000) mg, respectively.

**Table 8:** ATWB: Treatment discontinuation, corticosteroid treatment, and deaths Data represented as n (%); p value calculated by chi-square; *Any two or more of the above therapies (CTLA-4 inhibitors, PD-1 inhibitors, and PD-L1 inhibitors); †Any other antineoplastic treatment not belonging to the class of ICIs specifically listed. ATWB, elevated AT levels along with elevated bilirubin; CTLA-4, cytotoxic T-lymphocyte–associated protein 4; ICI, immune checkpoint inhibitors; ICI Comb, combinations of ICIs; n, patients with IAT; PD-1, programmed cell death 1; PD-L1, programmed cell death ligand 1.

(A) Treatment class (n)	(B) Treatment discontinued (% of A)	Treatment discontinued with death < 45 days (% of B)	(C) ATWB events treated with corticosteroids (% of A)	ATWB events treated with corticosteroids with death < 45 days (% of C)
All (441)	43 (9.75)	21 (48.84)	147 (33.33)	68 (46.26)
CTLA-4 inhibitors (16)	2 (12.50)	0	3 (18.75)	1 (33.33)
PD-1 inhibitors (277)	27 (9.75)	13 (48.15)	81 (29.24)	32 (39.51)
PD-L1 inhibitors (15)	1 (6.67)	1 (100.00)	5 (33.33)	5 (100.00)
ICI Comb^*^ (73)	5 (6.85)	2 (40.00)	34 (46.58)	16 (47.06)
ICI plus antineoplastic^† ^(60)	8 (13.33)	5 (62.50)	24 (40.00)	14 (58.33)
p value	0.7591	0.5139	0.0317	0.0106

Sensitivity Analyses

Results from a sensitivity analysis after exclusion of all patients with primary or secondary liver cancer were similar to those observed without excluding them. Additional sensitivity analysis removing treatment courses without any LFT elevation at baseline (< grade 1; approximately 18.6% of the total population) resulted in a slightly lower frequency of elevations (IAT: 1.63% and ATWB: 0.39%) than those observed in full study population (IAT: 2.11% and ATWB: 0.52%). Overall, the results were similar across all the data reported.

## Discussion

This retrospective study, which aimed to describe the frequency, onset, duration, and management of ≥ grade 3 elevated AT levels with or without elevated bilirubin, included 69,140 patients with solid tumors who underwent 85,433 treatment courses with ICIs, including monotherapy or combination of ICIs and ICIs plus antineoplastic agents. Approximately half of all patients with solid tumors had initiated treatment at an age ≥ 70 years, and approximately 40% had a diagnosis of lung cancer. Overall, the patients with lung cancer were older, with most patients being diagnosed between 65 and 74 years. The majority of patients were treated with ICIs, such as PD-1, PD-L1, and CTLA-4 inhibitors at the time of study inclusion. This finding is congruent with the results from a real-world evidence study that summarized the effects of PD-1/PD-L1 inhibitors in patients with non-small cell lung cancer and reported that these agents were more effective in patients aged ≥ 70 years than those aged < 70 years [25].

Patients with normal or with elevated (grade 1 or 2) LFTs prior to initiation of ICI treatment were included in the study. Sensitivity analyses suggested only small differences in the frequency of LFT events after excluding these patients with grade 1 or 2 baseline elevations. As the majority of patients had minor LFT elevations at baseline, this indicated that most physicians generally do not have concerns of patients with potential hepatic issues and was an important issue to evaluate. The occurrence of IAT in these patients occurred within all the different classes of ICIs. The reason to limit the study population with normal or grade 1 or 2 LFTs at baseline was to identify treatment-emergent ≥ grade 3 elevated AT levels. In this study, the proportion of IAT ranged from approximately 0.99%-6.81% among patients receiving treatment courses for solid tumors. For patients receiving ICI monotherapy for treatment of solid tumors, the incidence of IAT was 0.99%-3.01%. However, the incidence increased to approximately 6.81% in patients receiving combination therapy with ≥ two ICIs. Similar results have been reported in the literature, indicating that the occurrence of IAT is higher in patients treated with ICI combination therapy (6%-13%) than those treated with ICI monotherapy (0.6%-3%) for various cancer types [26-30].

In general, a higher rate of elevated AT levels is reported when ICIs are combined with traditional chemotherapy or targeted therapies than that with ICIs alone. Results from a phase 3 study in patients with previously untreated metastatic melanoma (n = 502) reported that the incidence of grade 3 or 4 elevated AT levels in patients receiving ipilimumab plus dacarbazine (18%-22%) was greater than that observed in patients receiving dacarbazine plus placebo group (approximately 1%) [31]. Similarly, results from a phase 1 study (n = 6) that examined concurrent administration of ipilimumab and vemurafenib reported that 67% of patients with metastatic melanoma experienced grade 3 elevated AT levels [32]. Furthermore, the combination of nivolumab with sunitinib or pazopanib resulted in an 18%-20% increase in the incidence of grade 3 or 4 elevated AT levels in patients with metastatic renal cell carcinoma [33]. These studies involved patients with metastatic disease, in which cancer cells can infiltrate liver, thereby triggering elevation of AT levels [34-36]. Similar outcomes were also observed in patients with non-metastatic disease [37-40], which could be hypothesized to occur due to additive effect of the individual ICI and chemotherapeutic or targeted agent. Thus, it can be suggested that treatment with an ICI and antineoplastic drug led to further increase in AT levels in patients with elevated AT levels at baseline. In this study, a smaller number of treatment courses were associated with liver cancer (1.47%) or liver metastasis (6.41%) at baseline. However, sensitivity analyses indicated that results of this study were not affected by inclusion of these patients.

The proportion of patients with elevated bilirubin levels along with elevated AT levels is relatively low [41]. In this study, the proportion of ATWB ranged from approximately 0.2%-1.4% among patients receiving treatment courses for solid tumors. Similar to that observed with IAT, the highest incidence of ATWB was observed in patients treated with combination therapy consisting of two or more ICIs.

Along with ICIs, IAT and ATWB were also reported in studies of other immuno-oncology therapies. Results from a phase 2 study (n = 189) that evaluated safety and efficacy of blinatumomab, a bispecific T-cell engager (BiTE®) molecule, reported that 6.9% of patients with relapsed/refractory acute myeloid leukemia (ALL) experienced ≥ grade 3 ALT elevation [42]. In a single-center, phase 2 study (n = 75) of tisagenlecleucel, an anti-CD19 CAR T-cell therapy, grade 3 ALT, AST, and bilirubin elevation was observed in four (5%), five (7%), and eight (11%) patients with B-cell ALL [43]. However, as these therapies are not commonly administered in an outpatient community clinic setting, we did not include these patients.

Although not statistically significant, the median onset of ATWB from the time of initiation of treatment in patients occurred earlier than that of IAT. Also, while most of the events involving elevations of AT levels have a relatively quick onset, some patients may experience gradual LFT elevations through long exposure to the ICIs. Although these results are in accordance with the published literature, which report the median onset of hepatotoxicity in ICI monotherapy as one to three months [44-49] with the present study median of 51 days and interquartile range of 27-98, only one patient in the current analysis had a delayed onset of IAT and ATWB that was greater than 1,200 days. Since individual causality was not assessed in the study, the longer period for onset of IAT and ATWB might not be due to ICI treatment. In a pooled analysis of three trials evaluating a combination of ipilimumab and nivolumab in patients with advanced melanoma, the onset of events involving elevations of AT levels occurred in 8.4 weeks [30]. Similarly, the median time of onset of hepatotoxicity was 52 (16-151) days in a retrospective study in patients with malignant melanoma treated with either ICI monotherapy or two ICIs [24].

According to the best practices developed by collaboration of the International Consortium for Innovation and Quality in Pharmaceutical Development and DILI experts from academia and regulatory agencies, the management of immune-mediated liver injury caused by ICIs not only involves temporary or permanent discontinuation of ICI treatment, but also treatment with corticosteroids, mycophenolate mofetil, and other agents, such as anti-thymocyte globulin, calcineurin inhibitors, and rituximab [50]. AT levels > 5x ULN (≥ grade 3 elevations) and total bilirubin levels ≥ 2x ULN warrants discontinuation of ICI treatment and initiation of treatment with corticosteroids, such as prednisolone or methylprednisolone [50]. Reports of transient elevation of AT levels by drugs are common in literature and FDA’s guidelines suggest that these events do not warrant immediate treatment discontinuation [9]. In our study, ICI treatment for solid tumors led to few severe cases of ATWB and most patients continued treatment. A small proportion of treatment courses were discontinued soon after IAT and ATWB events. 

In many cases, the transient and asymptomatic elevations of AT levels might be owing to hepatic adaptation or drug tolerance, where the LFTs normalize after treatment continuation [51]. Treatment discontinuations were uncommon in this study, with approximately 90% of treatment courses with events involving elevations of AT levels not leading to treatment discontinuations. Approximately one-third of IAT (37.30%) and ATWB (33.33%) events were treated with corticosteroids rather than discontinuing the ICI treatment. In contrast, results from a retrospective study in patients undergoing treatment with ICI (n = 414) indicated that all patients were treated with corticosteroids after discontinuation of immunotherapy [52]. It has been reported that corticosteroid treatment should be initiated in patients with AT levels > 5x ULN [53], while other management guidelines suggest that grade 2 IAT elevations lasting longer than one to two weeks should be treated with corticosteroids [54]. In this study, the median duration of IAT and ATWB events was approximately seven days, suggesting that fewer patients needed corticosteroid treatment. Additionally, it can be postulated that patients receiving corticosteroids may have had severe liver injury as 46% of patients with ATWB treated with corticosteroids died within 45 days of the event.

Furthermore, treatment with corticosteroids may reduce the efficacy of ICI. Results from a real-world study in PD-L1-naïve patients with advanced non-small cell lung cancer (n = 640) treated with a PD-L1 blocker demonstrated that pre-treatment with prednisone (≥ 10 mg) was associated with decreased overall response rate, progression-free survival, and overall survival [55]. Similar findings were reported in an observational study evaluating the effect of concomitant prednisone on the efficacy of PD-1 blockers [56]. In another retrospective study in patients (n = 128) with advanced melanoma treated with ICIs, it was reported that treatment with corticosteroids might not be necessary for management of DILI [57]. In oncology clinical trials, ≥ grade 3 elevations of LFTs with a duration of ≤ seven days are not typically defined as dose-limiting toxicity and do not lead to treatment discontinuation [58-62].

This study has a few limitations that should be acknowledged. Owing to the observational and descriptive nature of the study, causality assessments in individual patients were not performed and conclusive evidence linking drug treatment to elevated AT levels with or without bilirubin could not be established. In addition, measurements of ALT, AST, and bilirubin were limited to what was routinely performed in the clinics; consequently, there may have been differences in the actual time of onset and duration of elevations of AT levels than when it was described in this study because LFTs are less frequently assessed in general practice than in clinical trials. Additionally, full medical histories, including prior treatment with hepatotoxic agents, were not available in the dataset analyzed in this study. Another limitation of the study was the absence of histologic data in the EHR. Hence, the proportion of patients treated for hepatocellular carcinoma and those with cirrhosis or other liver abnormalities was not known. Along with ICIs, any underlying liver disease, infections, tumour progression, and co-administration of other medications may contribute to the onset of IAT and ATWB. The impact of these factors was not evaluated in this study. Furthermore, data on some orally administered corticosteroids may not be complete, including doses of corticosteroids administered. Hence, the proportion of patients treated with corticosteroids may be somewhat underestimated and the results in this study are likely to represent lower frequency of patients who receive corticosteroids for management of LFT elevations. Moreover, when a patient received corticosteroids within 15 days of LFT elevations, it was assumed to be for treatment of IAT and ATWB, although causality for treatment was not listed in the EHR. In the current study, it was observed that a majority of treatment courses that led to elevations of AT levels did not result in treatment discontinuation. However, these findings did not evaluate whether the dose of ICI was modified to prevent elevations of AT levels and eventually, discontinuation of treatment. Additionally, ICI treatment could have been discontinued due to causes other than IAT and ATWB. Since the EHR database does not report IAT and ATWB as the causes of ICI treatment discontinuation, it was postulated that IAT and ATWB led to ICI treatment discontinuation. Notwithstanding these limitations, the study was conducted in a large number of patients across several clinics who had undergone a large number of treatment courses involving ICIs. Management of IAT and ATWB by administration of corticosteroids resulted in fewer treatment discontinuations. Finally, since the exact day of death was not available in the database to maintain patient confidentiality, there was some imprecision in recording the timing of death. Along with IAT and ATWB, death could have occurred due to various reasons such as disease progression and comorbidities in these patients, which were not recorded in the database. Nonetheless, this limitation is unlikely to bias the results in a way that would invalidate the directionality of the observed data. Overall, these study results should be generalizable to patients treated in a community oncology clinic setting and may not be applicable to in-patient hospitalizations at academic centers.

## Conclusions

In summary, this study described the infrequency of elevation of AT with or without elevation of bilirubin in patients receiving ICIs for treatment of solid tumors. Overall, the frequency of elevations of AT levels with or without bilirubin was found to vary slightly with ICIs, and the duration of the events was ≤ seven days in the majority of cases. Corticosteroids were administered for management of IAT and ATWB in > 33% of patients and < 15% discontinued ICI treatment, eventually leading to a minimal disruption in treatment courses.
